# Surgical Aortic Valve Replacement Combined With Coronary Artery Bypass Grafting in a Patient With Progeria

**DOI:** 10.1016/j.jaccas.2025.104823

**Published:** 2025-08-27

**Authors:** Simon Anquetil, Mathieu Bignon, Emré Belli, Fabien Labombarda

**Affiliations:** a Department of Pediatric Cardiology, CHU de Caen, Caen, France; b Department of Cardiology, CHU de Caen, Caen, France; c Department of Pediatric and Adult Congenital Heart Diseases, Marie Lannelongue Hospital, Groupe Hospitalier Saint Joseph, Reference Center of Complex Congenital Heart Diseases M3C, Le Plessis Robinson, France; d Department of Cardiology and Pediatric Cardiology, CHU de Caen, Caen, France

**Keywords:** cardiovascular disease, coronary artery bypass, valve replacement

## Abstract

**Background:**

Calcific aortic stenosis is increasingly recognized as a major determinant of mortality in the aging subset of patients with the ultra-rare Hutchinson-Gilford progeria syndrome (HGPS), particularly as survival improves with lonafarnib therapy. However, the optimal treatment strategy for severe aortic stenosis in this population remains undefined.

**Case Summary:**

We report the first successful case of combined surgical aortic valve replacement and coronary artery bypass grafting in a severely symptomatic 21-year-old man with HGPS, severe calcific aortic stenosis, and coronary artery disease.

**Discussion:**

Despite anatomical and procedural challenges, surgical valve replacement represents a reasonable treatment option in patients with HGPS when transcatheter or alternative approaches are not feasible. A multidisciplinary approach is essential to optimize outcomes in this high-risk population.

**Take-Home Message:**

Surgical aortic valve replacement may be considered among the potential treatment options for selected patients with HGPS.

## Graphical Abstract

**Figure:**
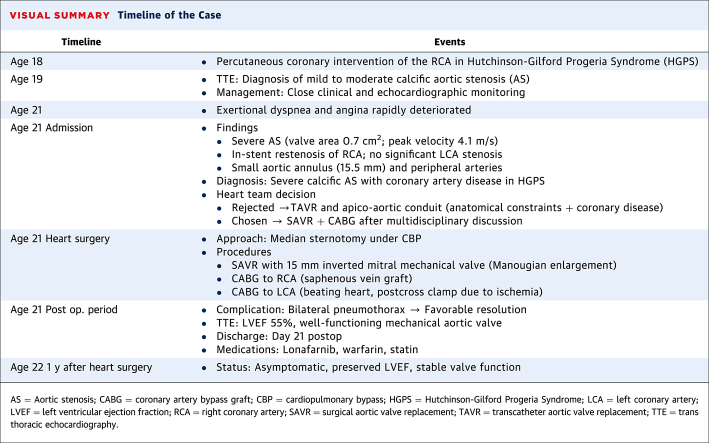

## History of Presentation

A 21-year-old man with Hutchinson-Gilford progeria syndrome (HGPS) was admitted for the evaluation and management of severe calcific aortic stenosis (AS) complicated by coronary artery disease. He reported progressively worsening exertional angina and shortness of breath over the preceding weeks.

**Take-Home Messages**

• SAVR could be considered as a potential option in selected cases of severe AS among patients with HGPS, particularly when less invasive approaches are not suitable.
• A multidisciplinary approach is essential to optimize outcomes in this uniquely high-risk population.

## Past Medical History

At 18 years of age, the patient underwent percutaneous coronary intervention for significant right coronary artery (RCA) stenosis. A year later, moderate calcific AS was diagnosed and closely monitored. By 21 years of age, the AS had progressed to severe symptomatic disease. The patient had no prior surgical history and was on lonafarnib therapy at the time of admission.

## Differential Diagnosis

The primary differential included severe calcific AS as the main cause of exertional angina and myocardial ischemia. Restenosis of the previously treated RCA and new significant stenosis of the left coronary artery (LCA) were also considered major contributors. Other possibilities, such as microvascular dysfunction or hypertensive cardiomyopathy, were deemed less likely given the imaging and clinical findings.

## Investigations

**Figure 1:**
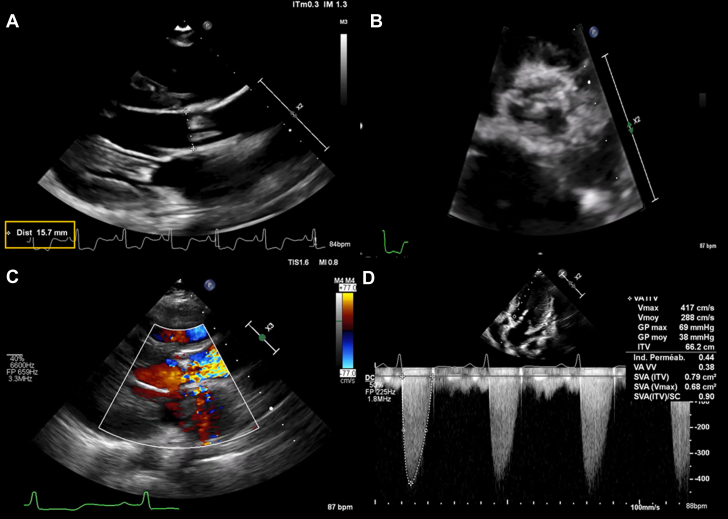
Transthoracic Echocardiography (A) Small aortic annulus (parasternal long-axis view). (B) Severe calcification of the aortic valve leaflets (parasternal short-axis view). (C) High-velocity jet through the aortic valve with a mosaic pattern indicating turbulent flow (color Doppler, parasternal long-axis view). (D) Doppler profile consistent with severe aortic stenosis (continuous-wave Doppler, apical 5-chamber view).

Electrocardiography revealed signs of myocardial ischemia and left ventricular hypertrophy. Troponin levels were within normal limits, but B-type natriuretic peptide was elevated at 496 pg/mL (normal value <100 pg/mL), raising concern for advanced calcific AS and ischemic burden. Transthoracic echocardiography (TTE) showed severe AS (peak velocity 4.1 m/s, valve area 0.7 cm^2^, mean gradient 39 mm Hg) with heavily calcified aortic valve leaflets, a small calcified annulus (diameter 15.5 mm), and mild central aortic regurgitation (Figure 1). The aortic root was small (sinuses 17 mm, tubular aorta 20 mm). The left ventricle was nondilated and hypertrophic (septal thickness 12 mm, posterior wall thickness 12 mm), with no regional wall motion abnormalities, preserved systolic function (ejection fraction 69%, Simpson's method), and diastolic dysfunction. The mitral valve was calcified with mild regurgitation. Both the mitral leaflets and the subvalvular apparatus were moderately calcified, but leaflet mobility was preserved and mitral regurgitation was only mild. There were no signs of systolic anterior motion or dynamic left ventricular outflow tract obstruction.

**Figure 2:**
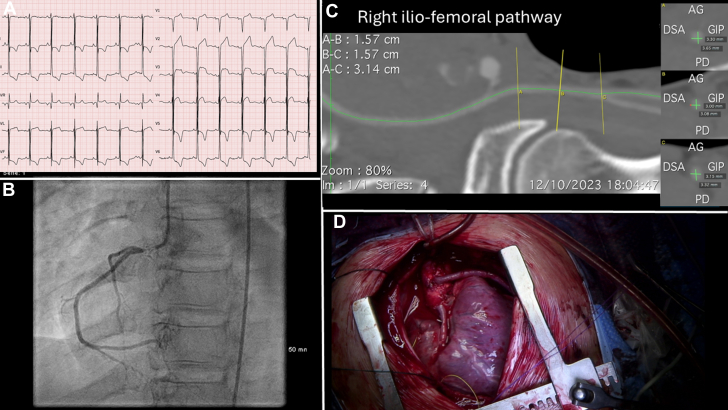
Preoperative and Intraoperative Findings (A) Electrocardiogram showing signs of left ventricular hypertrophy and repolarization abnormalities. (B) Coronary angiography showing in-stent restenosis of the right coronary artery. (C) Computed tomography angiography showing small right iliofemoral pathway. (D) Intraoperative view of coronary artery bypass grafts to the right and left coronary arteries.

Right ventricular function was preserved, without pulmonary hypertension. Coronary angiography confirmed severe in-stent restenosis of the RCA and no significant stenosis of the LCA. Computed tomography angiography revealed critically small iliofemoral arteries (Figure 2C).

## Management

Given the severe in-stent restenosis of the RCA, small aortic annulus, and limitations of transcatheter access, transcatheter aortic valve replacement (TAVR) was deemed unsuitable. After multidisciplinary evaluation, 2 surgical strategies were considered: an apicoaortic valved conduit or surgical aortic valve replacement (SAVR). SAVR was deemed feasible and preferred for its potential to address both valvular and coronary pathology. A median sternotomy was performed under cardiopulmonary bypass, with myocardial protection using antegrade intermittent warm blood cardioplegia. The aortic valve was replaced with a size 15 inverted mitral mechanical prosthesis following annular enlargement using the Manougian technique. Simultaneously, a saphenous vein graft harvested from the right leg was used for coronary artery bypass grafting (CABG) of the occluded RCA. We observed during cardioplegia administration that the unique patent left coronary ostium appeared relatively restrictive. Following aortic unclamping and myocardial reperfusion, ischemic electrocardiographic changes were noted despite adequate hemodynamic parameters. Given the small and surgically manipulated aortic root and annular region after Manouguian enlargement, we suspected compromised perfusion of the LCA. To mitigate the risk of ongoing ischemia, we decided to add a precautionary saphenous vein graft to the left anterior descending coronary artery on a beating heart under cardiopulmonary bypass (Figure 2).

## Outcome and Follow-Up

Postoperative recovery was complicated by bilateral pneumothorax, which resolved favorably. TTE demonstrated a good left ventricular function (ejection fraction 55%), left ventricular hypertrophy of a well-functioning prosthetic aortic valve (peak velocity: 2.0 m/s, mean gradient: 16 mm Hg), stable mitral valve function, and no features suggestive of “suicide ventricle” physiology. The patient was discharged on day 21 with ongoing treatment including lonafarnib, warfarin, and a statin. At 12-month follow-up, he remained asymptomatic with preserved left ventricular systolic function and a well-functioning prosthetic aortic valve.

## Discussion

HGPS is an ultra-rare genetic disease with a dramatically shortened lifespan subsequent to accelerated premature atherosclerosis and valvular calcifications.^1^ In the new older segment of the HGPS population treated with lonafarnib,^2^ AS is emerging as a principal determinant of mortality. In HGPS patients, AS progresses rapidly, reaching a critical stage within a few years of detecting aortic valve calcification, unlike in the general population. Therapeutic options for severe AS in HGPS patients present significant challenges, including anatomical constraints (such as small peripheral arterial calibers and aortic annulus sizes, which may limit valve options), high surgical risk (due to comorbidities and extreme frailty), complex anesthetic and postoperative management, and finally, the critical issue of timing of intervention. Consequently, standard SAVR was considered to be a very high-risk procedure, and alternative strategies, including TAVR and apicoaortic conduit, were proposed as alternative treatment options, both with their own limits and risks. The main critical points for the TAVR in patients with HGPS have been previously emphasized^3^: transapical access is required due to small femoral vessel diameter, the extensive calcification of the aortic valve and left ventricular outflow tract, the low position/proximity of the coronary ostia, the increased risk of aortic annulus rupture due to the tissue frailty that characterizes the progeria disease, and finally, the impossibility to propose TAVR in patients with very small aortic annulus diameter, as for our patient. Similarly, key considerations for an apicoaortic conduit include the necessity of cardiopulmonary bypass, the lack of appropriately small apicoaortic conduit systems on the market, the technical challenge of creating an apical defect in the left ventricle to accommodate the proximal conduit, and the potential inability to connect the conduit to the descending aorta due to extensive calcifications.^4^ Additionally, there is a risk of conduit compression or distortion due to the small chest cavity, particularly in the presence of a dilated left ventricle. Compared with these alternatives, SAVR enabled concurrent CABG, offering a more definitive solution for combined valvular and coronary pathology, which may help improve overall prognosis in these patients. To our knowledge, this is the first reported case of successful SAVR with CABG in a patient with HGPS. A prior case involving a 33-year-old patient with an adult progeroid condition, so-called Werner syndrome, supports the feasibility of surgical intervention in selected progeroid phenotypes.^5^ We considered the use of a homograft; however, this would have necessitated full root replacement with coronary reimplantation, which we judged to be high risk in this patient with fragile tissue. In addition, homografts in this size are not readily available. Similarly, no appropriately sized stentless valve was suitable for the very small annulus (15.5 mm). Given these limitations, we opted for a mechanical valve, as annular enlargement allowed implantation of a small mechanical valve, which provided optimal hemodynamic performance in this context. Although mechanical valves require lifelong anticoagulation, our patient had no contraindication to anticoagulation.

This report has limitations inherent to single-case experiences. However, it underscores the importance of individualized treatment planning in HGPS, highlights the limitations of conventional approaches such as TAVR in this rare population, and suggests that SAVR may be feasible in selected cases. Given the HGPS-specific risk factors and limited surgical experience in this population, a collaborative approach and careful multidisciplinary evaluation are essential for accurate risk stratification, optimal timing of intervention, and improved outcomes.

## Conclusions

SAVR combined with CABG can be successfully performed in selected patients with HGPS, offering a potential treatment option in this rare and uniquely high-risk population.

Written informed consent was obtained from the patient for publication of this case report.

**Visual Summary:** Timeline of the Case

Timeline	Events
Age 18	• Percutaneous coronary intervention of the RCA in Hutchinson-Gilford Progeria Syndrome (HGPS)
Age 19	• TTE: Diagnosis of mild to moderate calcific aortic stenosis (AS) • Management: Close clinical and echocardiographic monitoring
Age 21	• Exertional dyspnea and angina rapidly deteriorated
Age 21 Admission	• Findings • Severe AS (valve area 0.7 cm^2^; peak velocity 4.1 m/s) • In-stent restenosis of RCA; no significant LCA stenosis • Small aortic annulus (15.5 mm) and peripheral arteries • Diagnosis: Severe calcific AS with coronary artery disease in HGPS • Heart team decision • Rejected →TAVR and apico-aortic conduit (anatomical constraints + coronary disease) • Chosen → SAVR + CABG after multidisciplinary discussion
Age 21 Heart surgery	• Approach: Median sternotomy under CBP • Procedures • SAVR with 15 mm inverted mitral mechanical valve (Manougian enlargement) • CABG to RCA (saphenous vein graft) • CABG to LCA (beating heart, postcross clamp due to ischemia)
Age 21 Post op. period	• Complication: Bilateral pneumothorax → Favorable resolution • TTE: LVEF 55%, well-functioning mechanical aortic valve • Discharge: Day 21 postop • Medications: Lonafarnib, warfarin, statin
Age 22 1 y after heart surgery	• Status: Asymptomatic, preserved LVEF, stable valve function

AS = Aortic stenosis; CABG = coronary artery bypass graft; CBP = cardiopulmonary bypass; HGPS = Hutchinson-Gilford Progeria Syndrome; LCA = left coronary artery; LVEF = left ventricular ejection fraction; RCA = right coronary artery; SAVR = surgical aortic valve replacement; TAVR = transcatheter aortic valve replacement; TTE = trans thoracic echocardiography.

## Funding Support and Author Disclosures

The authors have reported that they have no relationships relevant to the contents of this paper to disclose.
